# Gene Regulatory Network Modeling of Macrophage Differentiation Corroborates the Continuum Hypothesis of Polarization States

**DOI:** 10.3389/fphys.2018.01659

**Published:** 2018-11-27

**Authors:** Alessandro Palma, Abdul Salam Jarrah, Paolo Tieri, Gianni Cesareni, Filippo Castiglione

**Affiliations:** ^1^Department of Biology, University of Rome Tor Vergata, Rome, Italy; ^2^Department of Mathematics and Statistics, American University of Sharjah, Sharjah, United Arab Emirates; ^3^Institute for Applied Computing, National Research Council of Italy, Rome, Italy; ^4^Data Science Program, Sapienza University of Rome, Rome, Italy; ^5^Fondazione Santa Lucia Istituto di Ricovero e Cura a Carattere Scientifico (IRCCS), Rome, Italy

**Keywords:** macrophage, differentiation, phenotype, model, gene regulating network, polarization, immune system

## Abstract

Macrophages derived from monocyte precursors undergo specific polarization processes which are influenced by the local tissue environment: classically activated (M1) macrophages, with a pro-inflammatory activity and a role of effector cells in Th1 cellular immune responses, and alternatively activated (M2) macrophages, with anti-inflammatory functions and involved in immunosuppression and tissue repair. At least three different subsets of M2 macrophages, namely, M2a, M2b, and M2c, are characterized in the literature based on their eliciting signals. The activation and polarization of macrophages is achieved through many, often intertwined, signaling pathways. To describe the logical relationships among the genes involved in macrophage polarization, we used a computational modeling methodology, namely, logical (Boolean) modeling of gene regulation. We integrated experimental data and knowledge available in the literature to construct a logical network model for the gene regulation driving macrophage polarization to the M1, M2a, M2b, and M2c phenotypes. Using the software GINsim and BoolNet, we analyzed the network dynamics under different conditions and perturbations to understand how they affect cell polarization. Dynamic simulations of the network model, enacting the most relevant biological conditions, showed coherence with the observed behavior of *in vivo* macrophages. The model could correctly reproduce the polarization toward the four main phenotypes as well as to several hybrid phenotypes, which are known to be experimentally associated to physiological and pathological conditions. We surmise that shifts among different phenotypes in the model mimic the hypothetical continuum of macrophage polarization, with M1 and M2 being the extremes of an uninterrupted sequence of states. Furthermore, model simulations suggest that anti-inflammatory macrophages are resilient to shift back to the pro-inflammatory phenotype.

## Author Summary

Macrophages are key players in the elicitation of an efficient immune response. Latest classification of macrophage functional types comprises the classically activated (M1) macrophages with a pro-inflammatory activity and the alternatively activated (M2) macrophages, with anti-inflammatory functions. The latter is further subdivided into at least three different subsets, namely, M2a, M2b, and M2c, which are characterized on the basis of distinct eliciting signals.

Accounting for the gene-related mechanisms of macrophage differentiation is a challenging task. We have used the methodology known as gene regulation network modeling on a newly constructed network of gene regulation originated from published experimental data. We have used computer simulations to explore the dynamical behavior of this network and derived conclusions about the hypothetical continuum of macrophage polarization with M1 and M2 being the extremes of an uninterrupted sequences of states. Our simulations also suggest that anti-inflammatory macrophages are resilient to shift to the pro-inflammatory phenotype.

## Introduction

**FIGURE 1:**
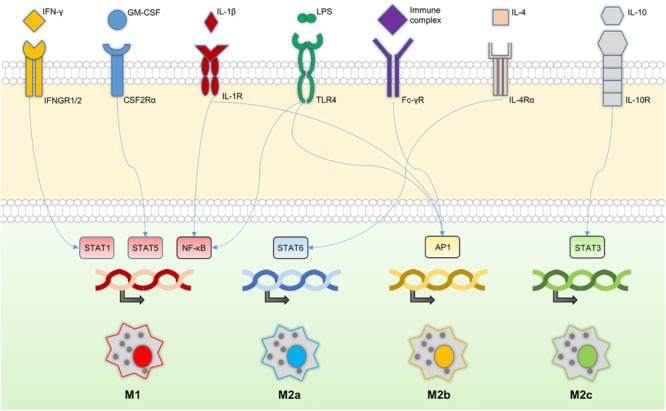
Macrophage signaling cascade. Macrophage receptors and their relationships with key transcription factors downstream of the signaling cascade. The transcriptions of different sets of genes lead to distinctive macrophage phenotypes; M1, M2a, M2b, and M2c.

Macrophages and neutrophils of the innate immune system represent the first line of defense against most common microorganisms. Indeed, macrophages can recognize and respond to a wide range of stimuli, expressing a great variety of surface and intracellular receptors that activate several signal transduction pathways and complex gene expression patterns. Macrophages respond to extracellular stimuli upon contact with different cell types *via* endocytic, phagocytic, and secretory functions (Figure 1). Their activity is modulated by contact synapsis established with proximal cellular and molecular entities, including microorganisms, chemical mediators, and other macrophages (Gordon et al., 2014).

The monocyte–macrophage differentiation pathway is known to exhibit plasticity and diversity (Mantovani et al., 2002; Bowdish et al., 2007; Gordon, 2008; Mantovani, 2008). Similar to the polarization process of helper T type 1 and 2 cells (Th1–Th2), two distinct polarized forms of macrophages have been recognized in the past: the classically activated (M1) macrophage phenotype and the alternatively activated (M2) macrophage phenotype (Biswas and Mantovani, 2010). Moreover, macrophages have also been observed in “M2-like” states, which share some features of both M1 and M2. Indeed, recent studies support the view that fully polarized macrophages (M1 and M2) are the extremes of a continuum of macrophage polarization (Mantovani, 2008). For example, various stimuli, such as immune complexes (IC) together with LPS or interleukin-1 beta (IL-1β), glucocorticoids, transforming growth factor-β (TGF-β), and interleukin-10 (IL-10), give rise to M2-like functional phenotypes that share properties with IL-4- or IL-13-activated macrophages [such as high expression of mannose receptor (MR) and IL-10, as well as TNFα, IL-1β, and IL-6] (Mantovani et al., 2004). Variations of the gene expression patterns corresponding to M1 or M2 are also found *in vivo* (e.g., in the placenta and embryo, and during helminthic infection, *Listeria* infection, obesity, and cancer) (Raes et al., 2005; Biswas et al., 2006; Kraakman et al., 2014).

The M1 and M2 phenotypes Kraakman et al., 2014 correspond to cell activation states driven by cytokines, which are typically secreted by Th1, Th2, and T-regulatory cells, but also basophils, mast cells, B lymphocytes, and eosinophils. The M1 phenotype is polarized by single or a combination of Th1 cytokines and pro-inflammatory mediators, including granulocyte-macrophage colony-stimulating factor (GM-CSF), tumor necrosis factor (TNF)-α, IL-6, IL-1β, IL-12, and various pathogen-associated molecules, such as lipopolysaccharide (LPS). By contrast, the M2 polarization is induced by macrophage colony-stimulating factor (M-CSF), IL-4 and IL-13, IC, IL-10, as well as glucocorticoid, TGFβ, and serotonin (Sang et al., 2015) (see Table 1).

**Table 1:** Summary of key molecules in macrophage polarization as taken from the literature.

	M1	M2A	M2B	M2C
Cytokines	IL-10, IL-1, IL-23, IL-1β, TNFα, IL-6, IL-18	IL-10, IL-12, IL-23, IL-1Ra	IL-10, IL-12, IL-23, IL-1β, TNFα, IL-6	IL-10, IL-12, IL-23, TGFβ
CC-chemokines	CCL-2, 3, 4, 5, 11, 17, 22	CCL-17, 18, 22, 24	CCL-1	CCL-16, 18
CXC-chemokines	CXCL-1, 2, 3, 5, 8, 9, 10, 11, 16	–	–	CXCL-13
Scavenger receptors	–	SR, MR	–	MR, CD163
Metabolism	iNOS	FIZZ-1, Ym-1, Arg	iNOS	Arg

*Cytokines, chemokines, receptors, and genes involved in metabolism are represented for each specific macrophage phenotype (adapted from Foey, 2014). Dashes indicate missing/contrasting data in the literature.*

Although there is a wealth of information about the different macrophage subsets *in vitro*, features such as plasticity, heterogeneity, and adaptability make them very difficult to study using conventional experimental tools. Furthermore, as many of the studies are done in different settings or for different goals, some literature reports are not conclusive and sometimes contradictory. It is not clear how robust the different macrophage subsets are to environmental changes. In particular, how does a modification of the cytokine environment affect the phenotype of macrophages? Which polarization state is most stable? Which possible gene knockouts can lead to a phenotypic change?

Macrophages polarization is essential in orchestrating the immune system response both in infectious and sterile immune settings. To shed light on this complex molecular process and address the questions above, we employed computational modeling of *gene regulatory networks* (GRNs) (Karlebach and Shamir, 2008).

Computational and mathematical modeling provide a means to assemble the known relevant molecules and their interactions into a network of pathways, with cross-talk between them. This allows, for examples, the test of whether the assimilated knowledge is sufficient to reproduce experimental results, and, furthermore, introduce cell-specific perturbations into the network to generate and test hypotheses *in silico*. For recent reviews, see Eftimie et al. (2016), Chakraborty (2017).

Computational models of GRNs have been shown to be a good approach to study how cells integrate several signals driving the cell phenotypic changes, especially for their ability to quantitatively and qualitatively describe a great variety of poorly characterized biological situations (Méndez and Mendoza, 2016). Computational models are used to describe immunological phenomena, to provide a better understanding of aspects of the immune response, and to produce outcomes coherent with available data, thus unraveling basic mechanisms of immunology and possibly leading to new hypotheses that can be tested experimentally *in vivo* or *in vitro* (Castiglione and Celada, 2015).

Discrete logical (Boolean or multi-state) models are usually the method of choice especially when the biological questions are of qualitative nature or when the available data (and knowledge) are mainly qualitative. Boolean networks and logical models have been used extensively to model many biological systems including immunological systems such as T-cell signaling and T helper cell differentiation (Naldi et al., 2010; Abou-Jaoudé et al., 2016; Méndez and Mendoza, 2016).

There are several computational models of some pathways that are involved in the pro and anti-inflammatory immune response, such as the NF-κB, TNF-α, IL-1, and IL-10 signaling pathways. Furthermore, there are computational models of T helper cell differentiation including continuous (Carbo et al., 2014), Boolean (Martinez-Sanchez et al., 2015), multistate logical (Naldi et al., 2010), and multi-scale (Santoni et al., 2008; Tieri et al., 2014). However, we are not aware of any GRN models of the molecular network describing macrophage differentiation. We have recently developed a multiscale model (Castiglione et al., 2016) of the immune response incorporating a minimalistic Boolean model of macrophages differentiation accounting for M1 and M2 polarization, but not for the subsets of M2. Maiti et al. (2014) presented an ODE model to describe the pro- and anti-inflammatory signaling in macrophages toward understanding immune homeostasis.

In this paper, we present a novel logical model of the gene regulation underlying macrophage differentiation and polarization, where the regulatory interactions and logical rules are inferred from the literature. We then used the model to study the dynamical behavior of the network. The model not only was able to reproduce known experimental data but also provides the first computational evidence of the continuum hypothesis of phenotypes which was suggested by Sica and Mantovani (2012).

## Materials and Methods

### Logical Models of Regulatory Networks

Gene regulatory network modeling aims at describing the way cells integrate extracellular stimuli to run cellular programs consisting of activations and inhibitions of genes (Kestler et al., 2008).

Logical network modeling was introduced by the geneticist R. Thomas (Thomas and D’Ari, 1990; Thomas and Kaufman, 2001) for the study of GRNs. Since then, they have been developed further, and have been used extensively to model many biological systems including cell-fate determination in *A. thaliana* (Espinosa-Soto et al., 2004; Benítez and Hejátko, 2013), *E. coli* metabolism (Samal and Jain, 2008), and the differentiation and plasticity of T helper cells (Naldi et al., 2010; Abou-Jaoudé et al., 2014), to name a few.

Gene regulatory networks are typically drawn from a mixture of literature, data mining and experimental data. Signal transducers, transcription factors and target genes in the activation of specific cellular programs (e.g., cell maturation or differentiation) are identified, as well as their relationships coded in terms of inhibition/activation. This data mining step produces a network (*N, E*) in which the nodes *N* are the molecules and the edges *E = E^-^* ∪*E^+^* are the activations (edges in the set *E^+^*) and inhibitions (edges in *E^-^*) relationships. Gene activation levels (states) or molecular concentrations are represented either by a discrete and usually very small set of values (two levels, i.e., active/inactive, represents the most used one, called Boolean) or by a continuous range of activity levels. In this paper, we have used the discrete Boolean formulation.

Each node *nk* of the network *N* has a function *F_k_* specifying how the state of that node may change in response to changes in the states of its neighbors (the nodes *nj* for which there exists an edge *ejk* ∈*E*) in the network. The synchronous or asynchronous calculation of the functions *F*_1_,…,*F_n_*, at each discrete step makes the network evolve from one macro-state to another. In the synchronous mode, all node states are updated at the same time, while in an asynchronous case, nodes are randomly updated at different time steps.

The Boolean model of a GRN is therefore defined as a discrete dynamical system which can then be studied for its dynamical properties. Since the space of all possible macro-states is finite, starting from any configuration, the repeated application of the functions *F*_1_,…,*F_n_*, will lead the system to be in states that it has reached before. These states correspond to stable patterns of gene expression that can be reasonably regarded as real biological states characterizing a specific cellular function. Starting from any configuration and after a certain transient period, the network dynamics will either reach a state and stay there (such a state is called a *steady state*), or can keep cycling forever among the same set of states (such a set of states is called a *limit cycle*) (Guevara, 2003; Ortiz-Gutiérrez et al., 2015). The transient period before the network dynamics reaches a certain steady state or limit cycle is called the basin of attraction of that state or cycle.

The dynamics of the system is encoded by a graph, whose vertices are all configurations (states) of the network and directed edges where each such edge indicates the transition of the system from one state to the next.

We used the software GINsim (Naldi et al., 2009) for the development of the model and the analysis of the network, including the identification of all steady states (Karlebach and Shamir, 2008; Méndez and Mendoza, 2016), and the BooleanNet Python library (Albert et al., 2008) and BoolNet R library (Müssel et al., 2010) for the study of the dynamics of the system.

## Results

### Molecular Basis of the Macrophage Polarization

During the inflammation process, several immune cells are involved in initiating and maintaining the inflammatory state. Macrophages, together with leukocytes, are the first cells recruited to the inflammation site. They start releasing pro-inflammatory cytokines (mostly IFN-γ and IL-1β), creating an inflammatory environment. The binding of those molecules to their specific receptors triggers a signal transduction cascade resulting in the release of other inflammatory molecules. This positive feedback mechanism allows the maintenance of the inflammatory state and reinforce the M1 polarized state.

The resolution of inflammation occurs by different mechanisms, such as the downregulation of pro-inflammatory molecules, the short half-life of the inflammatory mediators, and the production of anti-inflammatory molecules. In this context, macrophages are expected to switch to M2, and, consequently, produce anti-inflammatory mediators, such as IL-10, inhibiting M1-related transcriptional regulators, while a positive feedback loop provides the means to maintain their anti-inflammatory phenotype.

Interferon (IFN) receptors have multi-chain structures and interact with members of the Janus-activated kinase (JAK) family (Darnell et al., 1994). When IFN-γ binds to its cognate receptor, the activation of the receptor-associated JAKs occurs in response to rearrangement and dimerization of the receptor subunits, followed by auto-phosphorylation and activation of the associated JAKs. This process determines the activation of classical JAK–STAT (signal transducer and activator of transcription) signaling pathways, resulting in the transcription of target genes (Platanias, 2005; Mosser and Edwards, 2008; McLaren and Ramji, 2009). Among the STATs, a pivotal role is played by STAT1, which undergoes dimerization after its JAK-mediated tyrosine phosphorylation. Hence, STAT1–STAT1 homodimer binds to *cis* elements known as “gamma-activated sequences” (GAS) in the promoters of the genes encoding NOS2, the MHC class II transactivator (CIITA) and IL-12, among others (Darnell et al., 1994; Sadler and Williams, 2008; Lawrence and Natoli, 2011). The IFN-associated JAK/STAT pathway exerts its function in the regulation of several immune cells, including macrophages, with a great increase of IFN production, the synthesis of several cytokines, such as interleukins IL-1β, IL-6, IL-12, IL-18, IL-23, and TNF-α, and nitric oxide (NO), as well as reactive oxide intermediates (ROI) and enzymes required for tissue remodeling.

Toll-like receptors (TLRs) mediate the immune response to a great variety of infectious agents and facilitate transcription of many pro-inflammatory genes (Sheikh et al., 2014). LPS is a component of the Gram-negative bacteria cell wall and induces expression of a wide variety of genes that constitute the innate immune response to bacterial infections. LPS signals through TLR4 on the cell surface of many cell types, including macrophages (Kawai and Akira, 2010, 2011). Signaling through TLR4 induces rapid activation of two distinct intracellular signaling pathways: one is the MyD88-dependent pathway, which leads the cascade through interferon regulatory factor (IRF)-3, and the other is the MyD88-independent signaling pathway, which acts through TIR-domain-containing adapter-inducing interferon β (TRIF). These pathways converge to activate the transcription of NOS2; the inducible NO synthase (Kawai et al., 2001; Doyle et al., 2002).

The M1 phenotype can also result from differentiation in the presence of GM-CSF, with increased expression of IL-12 and pro-inflammatory cytokines, the ability to activate Th1 cell immune responses and decreased expression of IL-10 (Krausgruber et al., 2011).

M2 macrophages exhibit a functionally distinct phenotype to that of M1s, originally *via* the ability of IL-4 to induce MR expression, followed by IL-13, which is another Th2 cytokine. IL-4/IL-13 and TGFβ/IL-10 have been described to be associated with priming M2 macrophage subsets (M2a and M2c, respectively). The role of IL-4- and IL-13-mediated signaling in M2 macrophage polarization has been well established both *in vitro* and *in vivo* (Gordon, 2003; Martinez et al., 2009; Gordon and Martinez, 2010). Mice with a myeloid cell-specific knockout of IL-4 receptor-α (IL4Rα) were found to lack M2 macrophage development in mouse models of helminth infection and in Th2 cell-mediated inflammation, where IL-4 has a major role (Lawrence and Natoli, 2011). It is well established that IL-4 and IL-13 are associated with Th2-type responses, which have well-defined effects on macrophages, other cells and immune functions. IL-4 and IL-13 are produced particularly in allergic, cellular, and humoral responses to parasitic and extracellular pathogens. IL-4 and IL-13 upregulate expression of the MR and MHC class II molecules by macrophages, which stimulates endocytosis and antigen presentation, and they induce the expression of selective chemokines (Gordon, 2003; Gordon and Martinez, 2010). IL-4 and IL-13 act through a common receptor chain – IL-4Rα – through signal transducer and activator of transcription 6 (STAT6).

Interleukin-1 beta and IC, together with TLR4-signaling inducers (i.e., LPS), drive the macrophage to an M2b phenotype. IL-1β not only plays a pivotal role in the initiation and maintenance of the inflammatory response but also modulates immunosuppressive mechanisms through the process of macrophages endotoxin tolerance. IL-1β is also produced in response to LPS, emphasizing a collaborative interplay between M1 and M2b macrophages in eliciting and maintaining the inflammatory response (Sato et al., 2012).

Interleukin-10 acts on a distinct plasma membrane receptor to those for IL-4 and IL-13 (Riley et al., 1999; Moore et al., 2001; Deng et al., 2012), and its effects on macrophage gene expression are different, involving a more profound inhibition of a range of antigen-presenting and effector functions, together with the activation of selected genes or functions. T cells themselves are more heterogeneous than was thought originally, including not only Th0-, Th1-, and Th2-type cells but also regulatory and possibly Th3-type cells, some of which secrete TGF-β and IL-10 (Gordon, 2003). TGFβ and IL-10 have been described to be associated with priming M2-like macrophages subset polarization. TGFβ and IL-10 modulate macrophage polarization and functional plasticity to that of an M2c subset which exhibits a characteristic cytokine phenotype of IL-10^hi^, IL-12^lo^, IL-23^lo^, and TGFβ^+^ which is associated with anti-inflammatory responses, scavenging, immune regulation, tissue repair, and tumor promotion. Both TGFβ and IL-10 directly suppress immune activation *via* the down-regulation of the expression of MHC II and pro-inflammatory cytokine production, with an indirect effect through cross-regulation of M1-derived cytokines and functionality (Gordon and Martinez, 2010; Lawrence and Natoli, 2011; Sica and Mantovani, 2012). IL-10 is a potent STAT3-dependent inhibitor of pro-inflammatory cytokine production and NO release, after challenge with LPS. IL-10-deficient mice develop widespread inflammatory cell infiltrates, including in the bowel, and transgenic animals that constitutively overexpress IL-metricconverterProductID10 in10 in macrophages suffer from septic shock and over-activity of pro-inflammatory cytokines (Lang et al., 2002b). The upregulation of expression of IL-4Rα by IL-10 correlates with increased IL-4-dependent expression of arginase-1. IL-10 also synergizes with LPS to increase the expression of arginase-2. Therefore, IL-10 increases the total level of arginases in macrophages in many ways (Lang et al., 2002a,b).

Phenotypes depending on complex regulatory logic can be effectively studied by using mathematical and computational approaches, such as GRN models.

### A Logical Network Model of Macrophage Differentiation

**FIGURE 2:**
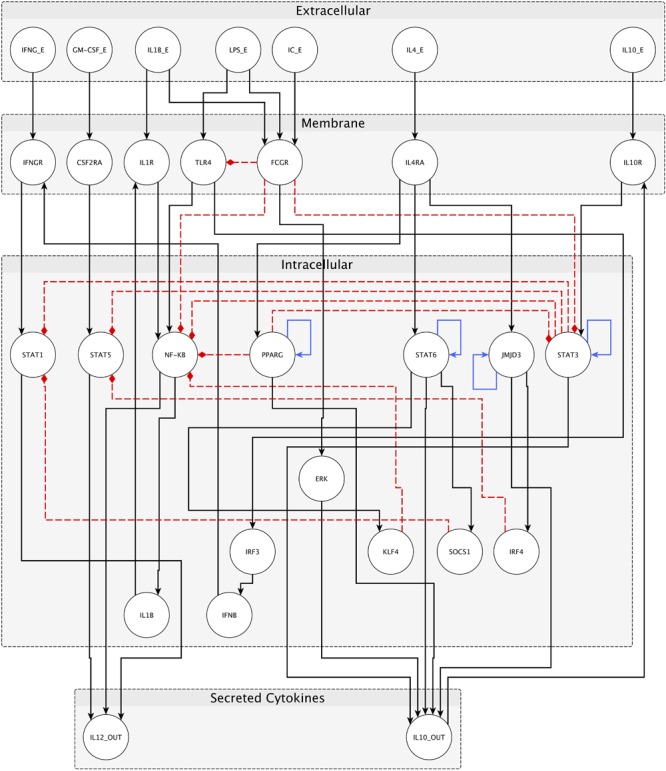
Network for macrophage polarization. External stimuli are reported in the extracellular space, receptors inside membrane space, and internal transducers/transcription factors in the intracellular space. Secreted cytokines (IL-10 and IL12) are also reported. Black arrows represent positive interactions (activations), red dashed arrows are negative interactions (inhibitions), and blue arrows are transcriptional auto-regulatory loops. Nodes represent both genes and proteins; edges represent both protein–protein interactions and transcriptional regulations.

We have constructed a logical regulatory network model (Figure 2 and Supplementary File S1) that describes macrophage polarization using experimental data and knowledge derived from literature (see Table 2) and a curated database of causal relationships between biological entities (Perfetto et al., 2016). The network comprises 30 nodes and 49 interactions among them. Interactions can be either positive (activations) or negative (inhibitions) (Figure 2). Table 2 shows a list of the molecules, interactions, and references from the literature supporting each interaction, while Table 3 shows logical rules for each molecule.

**Table 2:** Interactions in the macrophage polarization network.

Source	Interaction type	Target	Reference		Source	Interaction type	Target	Reference
IFNg_e	Positive	IFNgR	Kotenko et al., 1995; Mosser and Edwards, 2008; McLaren and Ramji, 2009		NF-κB	Positive	IL12_out	Tran-Thi et al., 1995; Lehtonen et al., 2002; Park et al., 2009; Lawrence and Natoli, 2011; Bally et al., 2015
IL1b_e	Positive	IL1R	Weber et al., 2010		NF-κB	Positive	IL1b	Tran-Thi et al., 1995; Lehtonen et al., 2002; Park et al., 2009; Lawrence and Natoli, 2011; Bally et al., 2015
GM-CSF_e	Positive	CSF2Ra	Lehtonen et al., 2002; Hamilton, 2008; Krausgruber et al., 2011; Lawrence and Natoli, 2011		PPARg	Positive	IL10_out	Ricote et al., 1998; Bouhlel et al., 2007; Lawrence and Natoli, 2011
LPS_e	Positive	TLR4	Park et al., 2009; Lawrence and Natoli, 2011					
LPS_e	Positive	FcgR	Nimmerjahn and Ravetch, 2008; Foey, 2014		PPARg	Negative	NF-κB	Ricote et al., 1998; Bouhlel et al., 2007; Lawrence and Natoli, 2011
IC_e	Positive	FcgR	Sánchez-Mejorada and Rosales, 1998; Nimmerjahn and Ravetch, 2008; Foey, 2014		PPARg	Negative	STAT3	Ricote et al., 1998; Bouhlel et al., 2007; Lawrence and Natoli, 2011
IL1b_e	Positive	FcgR	Nimmerjahn and Ravetch, 2008; Foey, 2014		STAT6	Positive	KLF4	Sica and Mantovani, 2012
IL4_e	Positive	IL4Ra	Gordon, 2003; Gordon and Martinez, 2010; Lawrence and Natoli, 2011					
IL10_e	Positive	IL10R	Moore et al., 2001; Foey, 2014; Hutchins et al., 2013; Nakamura et al., 2015		STAT6	Positive	SOCS1	Baker et al., 2009; Dickensheets et al., 2007; Whyte et al., 2011
IFNgR	Positive	STAT1	Mosser and Edwards, 2008; McLaren and Ramji, 2009		STAT6	Positive	IL10_out	Lang et al., 2002a; Gordon, 2003; Gordon and Martinez, 2010; Lawrence and Natoli, 2011
CSF2Ra	Positive	STAT5	Barahmand-Pour et al., 1998; Lehtonen et al., 2002; Hamilton, 2008; Krausgruber et al., 2011; Lawrence and Natoli, 2011		JMJD3	Positive	IRF4	Gordon, 2003; Ishii et al., 2009; Gordon and Martinez, 2010; Satoh et al., 2010; Lawrence and Natoli, 2011
IL1R	Positive	NF-κB	Weber et al., 2010		STAT3	Positive	IL10_out	Riley et al., 1999; Ritter et al., 1999; Hutchins et al., 2013; Foey, 2014; Nakamura et al., 2015
TLR4	Positive	IRF3	Sheikh et al., 2014					
TLR4	Positive	NF-κB	Tran-Thi et al., 1995; Lehtonen et al., 2002; Park et al., 2009; Lawrence and Natoli, 2011; Bally et al., 2015		STAT3	Negative	NF-κB	Riley et al., 1999; Hutchins et al., 2013
FcgR	Positive	ERK	Sánchez-Mejorada and Rosales, 1998; Sutterwala et al., 1998; Lucas et al., 2005; Nimmerjahn and Ravetch, 2008; Zhang et al., 2009; Luo et al., 2010; Clatworthy et al., 2014; Foey, 2014; Vogelpoel et al., 2014, 2015		STAT3	Negative	STAT1	Ito et al., 1999
FcgR	Negative	NF-κB	Sánchez-Mejorada and Rosales, 1998; Sutterwala et al., 1998; Ji et al., 2003; Lucas et al., 2005; Hirano et al., 2007; Nimmerjahn and Ravetch, 2008; Zhang et al., 2009; Luo et al., 2010; Clatworthy et al., 2014; Guilliams et al., 2014; Vogelpoel et al., 2014		STAT3	Negative	STAT5	Yamaoka et al., 1998
FcgR	Negative	STAT3	Sánchez-Mejorada and Rosales, 1998; Sutterwala et al., 1998; Ji et al., 2003; Lucas et al., 2005; Nimmerjahn and Ravetch, 2008; Zhang et al., 2009; Luo et al., 2010; Clatworthy et al., 2014; Guilliams et al., 2014; Vogelpoel et al., 2014, 2015		IRF3	Positive	IFNb	Doyle et al., 2002; Honda et al., 2005; Rauch et al., 2013; Mao et al., 2015
FcgR	Negative	TLR4	Sánchez-Mejorada and Rosales, 1998; Sutterwala et al., 1998; Abrahams et al., 2000; Nimmerjahn and Ravetch, 2008; Zhang et al., 2009; Luo et al., 2010; Guilliams et al., 2014; Vogelpoel et al., 2014, 2015		ERK	Positive	IL10_out	Sánchez-Mejorada and Rosales, 1998; Lucas et al., 2005; Nimmerjahn and Ravetch, 2008; Liu et al., 2009; Foey, 2014
IL4Ra	Positive	PPARg	Gordon, 2003; Bouhlel et al., 2007; Chawla, 2010; Gordon and Martinez, 2010; Gong et al., 2012		KLF4	Negative	NF-κB	Sica and Mantovani, 2012
IL4Ra	Positive	STAT6	Gordon, 2003; Ishii et al., 2009; Gordon and Martinez, 2010; Satoh et al., 2010; Lawrence and Natoli, 2011		SOCS1	Negative	STAT1	Dickensheets et al., 2007; Baker et al., 2009; Whyte et al., 2011
IL4Ra	Positive	JMJD3	Gordon, 2003; Ishii et al., 2009; Gordon and Martinez, 2010; Satoh et al., 2010; Lawrence and Natoli, 2011		IRF4	Negative	STAT5	Sica and Mantovani, 2012
IL10R	Positive	STAT3	Riley et al., 1999; Ritter et al., 1999; Hutchins et al., 2013; Foey, 2014; Nakamura et al., 2015					
STAT1	Positive	IL12_out	Mosser and Edwards, 2008; Sadler and Williams, 2008; McLaren and Ramji, 2009; Lawrence and Natoli, 2011		IFNb	Positive	IFNgR	Kotenko et al., 1995; Lehtonen et al., 2002; Gordon, 2003; Platanias, 2005; Lawrence and Natoli, 2011; Rauch et al., 2013
STAT5	Positive	IL12_out	Yamaoka et al., 1998; Lehtonen et al., 2002; Hamilton, 2008; Krausgruber et al., 2011; Lawrence and Natoli, 2011					

*Source and target nodes are reported as well as the sign of the interaction between them (positive: source molecule activates target molecule; negative: source molecule inhibits target molecule) and the references. Each input node is annotated with an “_e” suffix, which stands for external stimulus, as well as an “_out” suffix which stands for output.*

**Table 3:** Boolean functions in the macrophage polarization network.

Node	Boolean function	Reference
IFNgR	IFNg_e ∨ IFNb	Interferons bind to their cognate receptors (Kotenko et al., 1995; Lehtonen et al., 2002; Gordon, 2003; Platanias, 2005; Mosser and Edwards, 2008; McLaren and Ramji, 2009; Rauch et al., 2013)
CSF2Ra	GM-CSF_e	GM-CSF ligand binds to its receptor (Lehtonen et al., 2002; Hamilton, 2008; Krausgruber et al., 2011; Lawrence and Natoli, 2011)
IL1R	IL1b_e ∨ IL1b	IL-1 beta binds to its receptor (Weber et al., 2010)
TLR4	LPS_e ∧⌝ FcgR	TLR4 is activated by LPS; TLR4 signaling is inhibited by Fc gamma receptor (Sánchez-Mejorada and Rosales, 1998; Sutterwala et al., 1998; Nimmerjahn and Ravetch, 2008; Park et al., 2009; Zhang et al., 2009; Luo et al., 2010; Lawrence and Natoli, 2011; Vogelpoel et al., 2014)
FcgR	(IC_e ∧ LPS_e) ∨ (IC_e ∧ IL1b_e)	Immune complexes, together with LPS or IL-1 beta activate Fc gamma receptor (Sánchez-Mejorada and Rosales, 1998; Sutterwala et al., 1998; Abrahams et al., 2000; Lucas et al., 2005; Nimmerjahn and Ravetch, 2008; Zhang et al., 2009; Luo et al., 2010; Clatworthy et al., 2014; Guilliams et al., 2014; Vogelpoel et al., 2014, 2015)
IL4Ra	IL4_e	IL-4 binds to its receptor (Gordon, 2003; Gordon and Martinez, 2010; Lawrence and Natoli, 2011)
IL10R	IL10_e ∨ IL10_out	IL-10 binds to its receptor (Moore et al., 2001; Hutchins et al., 2013; Foey, 2014; Nakamura et al., 2015)
STAT1	IFNgR ∧ ⌝(SOCS1 ∨ STAT3)	Interferon-gamma receptor activates JAK/STAT1 pathway and is inhibited by SOCS1 or STAT3 signaling (Ito et al., 1999; Dickensheets et al., 2007; Mosser and Edwards, 2008; Baker et al., 2009; McLaren and Ramji, 2009; Whyte et al., 2011)
STAT5	CSF2Ra ∧ ⌝(STAT3 ∨ IRF4)	STAT5 transcription factor is activated *via* CSF2Ra signaling and inhibited by STAT3 or IRF4 (Barahmand-Pour et al., 1998; Ito et al., 1999; Lehtonen et al., 2002; Dickensheets et al., 2007; Hamilton, 2008; Baker et al., 2009; Krausgruber et al., 2011; Lawrence and Natoli, 2011; Whyte et al., 2011)
NF-κB	(IL1R ∨ TLR4) ∧ ⌝(STAT3 ∨ FcgR ∨ PPARg ∨ KLF4)	NF-κB transcription factor is activated by LPS or IL1-beta signaling cascades and inhibited by M2a- or M2b-related pathways (Tran-Thi et al., 1995; Ricote et al., 1998; Sánchez-Mejorada and Rosales, 1998; Sutterwala et al., 1998; Riley et al., 1999; Lehtonen et al., 2002; Bouhlel et al., 2007; Nimmerjahn and Ravetch, 2008; Park et al., 2009; Zhang et al., 2009; Luo et al., 2010; Weber et al., 2010; Lawrence and Natoli, 2011; Sica and Mantovani, 2012; Hutchins et al., 2013; Guilliams et al., 2014; Vogelpoel et al., 2014; Bally et al., 2015)
PPARg	IL4Ra	PPARg is activated by IL4 signaling (Gordon, 2003; Bouhlel et al., 2007; Chawla, 2010; Gordon and Martinez, 2010; Gong et al., 2012)
STAT6	IL4Ra	JAK/STAT6 pathway is activated by IL4 receptor after IL-4 binding (Gordon, 2003; Ishii et al., 2009; Gordon and Martinez, 2010; Satoh et al., 2010; Lawrence and Natoli, 2011)
JMJD3	IL4Ra	JMJD3 is activated in response to IL4 signaling cascade (Gordon, 2003; Ishii et al., 2009; Gordon and Martinez, 2010; Satoh et al., 2010; Lawrence and Natoli, 2011)
STAT3	IL10R∧ ⌝(FcgR ∨ PPARg)	JAK/STAT3 pathway is activated in response to IL-10 and inhibited by PPAR gamma or Fc gamma receptor pathways (Ricote et al., 1998; Sánchez-Mejorada and Rosales, 1998; Sutterwala et al., 1998; Riley et al., 1999; Ji et al., 2003; Bouhlel et al., 2007; Nimmerjahn and Ravetch, 2008; Lawrence and Natoli, 2011; Hutchins et al., 2013; Foey, 2014; Nakamura et al., 2015)
IRF3	TLR4	IRF3 is activated in response to TLR4 signaling pathway (Doyle et al., 2002; Sheikh et al., 2014; Mao et al., 2015)
ERK	FcgR	ERK pathway is initiated in response to M2b-related signals (Sánchez-Mejorada and Rosales, 1998; Lucas et al., 2005; Nimmerjahn and Ravetch, 2008; Liu et al., 2009; Foey, 2014)
KLF4	STAT6	KLF4 is activated downstream JAK/STAT6 pathway (Sica and Mantovani, 2012)
SOCS1	STAT6	SOCS1 is activated by STAT6 transcription factor (Baker et al., 2009; Whyte et al., 2011; Arnold et al., 2014)
IRF4	JMJD3	IRF4 is activated by JMJD3 expression (Gordon, 2003; Ishii et al., 2009; Gordon and Martinez, 2010; Satoh et al., 2010; Lawrence and Natoli, 2011)
IL1b	NF-κB	NF-κB transcription factor promotes IL-1 beta production (Tran-Thi et al., 1995; Lehtonen et al., 2002; Park et al., 2009; Lawrence and Natoli, 2011; Bally et al., 2015)
IFNb	IRF3	IRF3 promotes type I interferon production (Doyle et al., 2002; Honda et al., 2005; Rauch et al., 2013; Mao et al., 2015)
IL12_out	STAT1 ∨ STAT5 ∨ NF-κB	IL-12 is produced by transcription factors STAT1, STAT5 or NF-κB (Mosser and Edwards, 2008; Sadler and Williams, 2008; McLaren and Ramji, 2009; Lawrence and Natoli, 2011)
IL10_out	PPARg ∨ STAT6 ∨ JMJD3 ∨ STAT3 ∨ ERK	PPAR gamma, STAT6, JMJD3, STAT3 and ERK downstream genes lead to the production of high quantities of IL10 (Ricote et al., 1998; Sutterwala et al., 1998; Riley et al., 1999; Ritter et al., 1999; Lang et al., 2002a; Gordon, 2003; Lucas et al., 2005; Bouhlel et al., 2007; Ishii et al., 2009; Liu et al., 2009; Gordon and Martinez, 2010; Luo et al., 2010; Satoh et al., 2010; Lawrence and Natoli, 2011; Foey, 2014; Sanin et al., 2015)

*Based on the available literature (third column), a Boolean function (second column) is associated to each target node of the network (symbols ∧, ∨, and ⌝ indicate logical operators AND, OR, NOT, respectively).*

Nodes are of four kinds, depending on cellular location and function (Figure 2): seven *input* nodes, which represent the extracellular stimuli (IFNγ, GM-CSF, IL-1β, LPS, IC, IL-4, and IL-10), seven *receptors* (IFNγR, CSF2Ra, IL-1R, TLR4, FcγR, IL-4R, and IL-10R), 14 internal regulators (STAT1, STAT5, NF-κB, PPARγ, STAT6, JMJD3, STAT3, IRF3, ERK, KLF4, SOCS1, IRF4, IL1β, and IFN-β), and two main *products* of each distinct type of macrophage (IL-12 and IL-10). The input nodes represent the main intercellular molecular stimuli that drive macrophage polarization, as reported in the literature. Each external molecule (input) is connected to its specific receptor, and this binding elicits a signaling cascade, involving intracellular transducers and transcription factors (mostly STAT factors). Each specific transcription factor binds the promoter of a target gene, resulting in the production of IL12 or IL10 depending on the macrophage polarized form.

Interactions among nodes are derived from experimental data available in the literature as shown in Table 2. All interactions have been deposited in SIGNOR (Perfetto et al., 2016), a public database of causal interactions between biological entities. Each node is associated to a logical function which determines the activation level of the node based on the activation levels reached by its source nodes in the previous time step. The logical function of each node is inferred from the available literature (see Table 3).

The network encompasses several pathways. Different cell fates, i.e., macrophage phenotypes, are defined by *steady* or *stable states* (also called *fixed point attractors*) of gene expression, and described in this dynamic model as multiple, specific, and stable configurations of activated/deactivated nodes. In other words, stable states are configurations toward which the system tends to evolve, for a wide range of starting conditions. Thus, according to the network, its starting configuration, and the initial external stimuli, the pathways lead to a configuration that resembles a specific cell state in terms of the given gene expression pattern. In this regard, we assumed that the sum of the sizes of the basins of attraction of the steady states characterizes the likelihood of finding the cell in a specific differentiation state. In other words, the probability that the cell, stimulated by cytokines, will switch to the certain differentiation state is proportional to the size of the subspace of all network configurations eventually reached by the network dynamics.

Inhibitory pathways among M1 and M2 phenotype-related transcription factors are particularly interesting, because they allow a mutual exclusivity of transcription factors and, therefore, of the macrophage phenotypes, as reported in literature (Lawrence and Natoli, 2011). Notably, among the interactions describing the network and reported in tables above, the inhibition of TLR4 and NF-κB signaling by FcγR activation were added. These relationships allow the inhibition of M1 polarization in the presence of IC, that together with LPS and IL-1β, drives the otherwise absent M2b polarization.

To analyze the dynamics of the network under different conditions we used GINsim [Gene Interaction Network simulation^1^; (Chaouiya et al., 2012)], a software tool for modeling and simulation of genetic regulatory networks (Chaouiya et al., 2012). In some cases, for further confirmation or additional details, we used the BooleanNet Python (Albert et al., 2008) as well as the BoolNet R library (Müssel et al., 2010).

The fate of a macrophage strongly depends on the local biochemical microenvironment. To reproduce these different microenvironments that influence the cells, we defined a set of inputs to run the simulations. Hence, we could discriminate among steady states with a real biological meaning.

The starting expression state of the network corresponds to the naïve macrophage M0 (unstimulated/not-activated) phenotype, in which the state of each node in the network is set to “0” (i.e., low expression).

**FIGURE 3:**
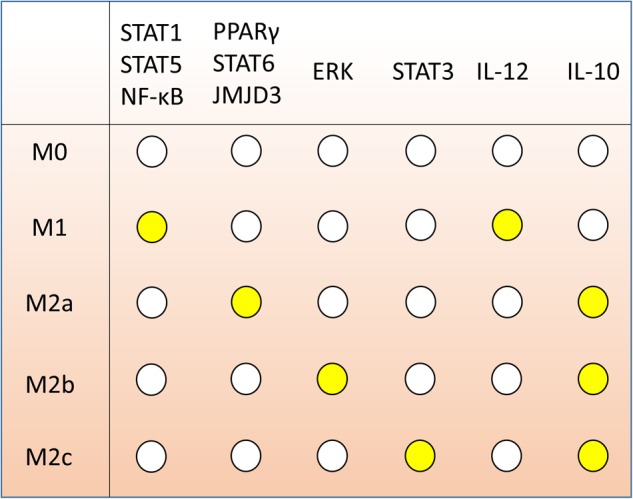
Gene expression markers of macrophage polarization according to literature. Each row, associated to one of M0, M1, M2a, M2b, and M2c, indicates the expression of the 10 marker genes determining the polarization fate. White dots represent inactive genes; yellow dots indicate expressed genes.

In our simulations, we found that our model has five sets of steady states fitting the following five specific macrophage phenotypes markers according to literature (Figure 3):

1. M0: no nodes active;
2. M1: IL-12 and at least one among STAT1, STAT5 or NF-κB are active;
3. M2a: all of PPARγ, STAT6, JMJD3 and IL-10 are active;
4. M2b: ERK and IL-10 are active; and
5. M2c: STAT3 and IL-10 are active.

**FIGURE 4:**
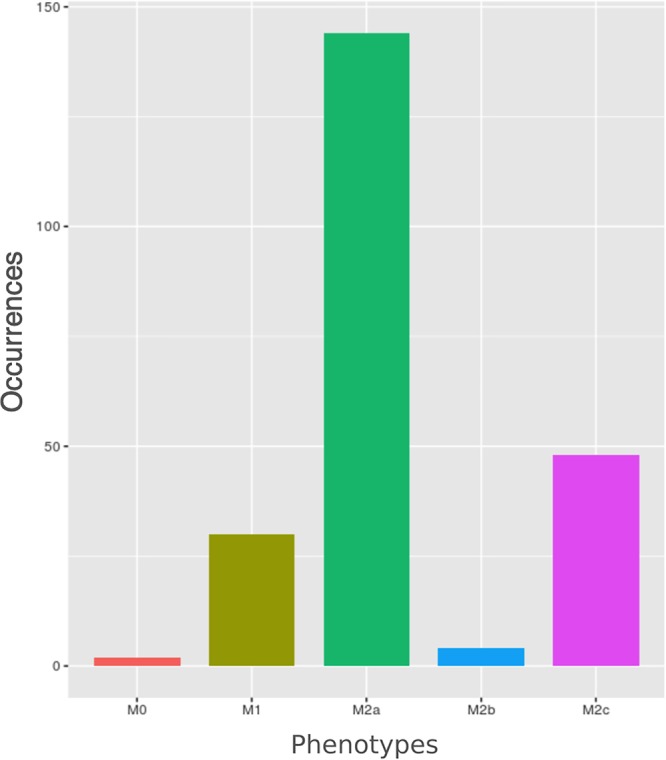
Barplot of macrophages’ phenotypes occurrences. Each bar represents the number of steady states (total number = 228) related to a specific polarized form.

We computed the steady states of macrophage polarization network using a synchronous update. The system reached 1056 states, 1040 of which are steady states and 16 are cycles made of three different states. Among the 1040 unique steady states (Supplementary Table S1), 228 can be mapped to the five *canonical* macrophage phenotypes reported *via* experimental studies in the literature. The frequencies of these 228 steady states are reported in Figure 4. The remaining steady states do not characterize the macrophage in any of the known canonical phenotypes. These states, for which there is a lack of experimental knowledge, could correspond to input conditions not existing among *in vivo* inflammation settings or even be artefacts of the modeling approach. Alternatively, they could correspond to *hybrid* phenotypes (O’Carroll et al., 2013) resembling gene expression patterns of two or more canonical phenotypes (discussed below). It is worth to note that a higher number of steady states does not imply a corresponding higher probability of polarization, since the final outcome depends on the combination of external stimuli. In other words, the number of steady states indicates the propensity of the network logic to lead the cell to the specific phenotypes yet driven by environmental cues.

The most frequent polarized state is the M2a followed by M2c and then M1. This is consistent with the pivotal role of macrophages in inflammation (M1), and in the resolution of inflammation (M2a and M2c). On the other hand, according to our analysis, M2b is the least frequent state, which might be consistent with the lack of knowledge of M2b-related pathways which is reflected in the network. This behavior of the model is consistent with observed data (Sica and Mantovani, 2012).

**FIGURE 5:**
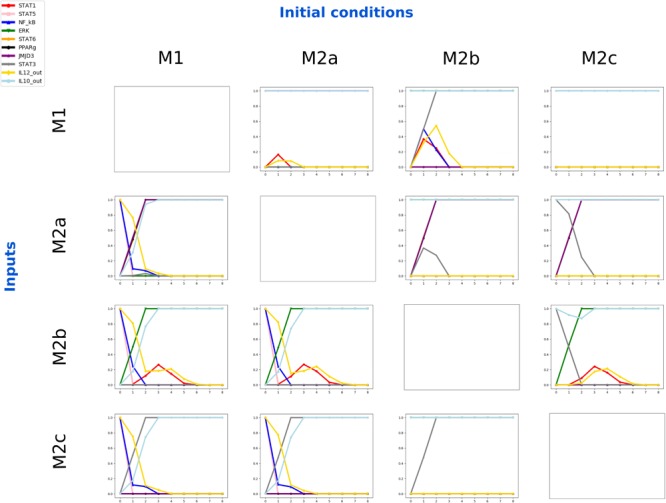
Dynamics of the gene activation levels obtained for all combinations of initial polarization state and polarizing stimuli. The average activation values are computed over 10^4^ asynchronous simulations of the activation level of the genes. For each subplot, the horizontal axis represents eight time steps and the vertical axis the average activity of a molecule from 0 to 1.

A closer look at the dynamics of the model (Figure 5) is obtained by performing several rounds of asynchronous simulations by using the BooleanNet Python library. We observed that any combination of stimuli among IFN-γ, IL-1β, LPS, and GM-CSF keep the polarization of the M1 macrophage. Once macrophages have polarized into an M1 form, the steady states are taken as initial conditions to polarize macrophages into the three different forms of M2 macrophage. IL-4 input is activated (i.e., IL-4 binding by IL-4RA) to polarize M2a macrophage, IL-10 is activated to polarize the M2c macrophages, and IC in combination with either IL-1β or LPS is activated to polarize M2b macrophages, according to the available literature on macrophage polarization stimuli (Gordon and Martinez, 2010).

The M1 polarization is simulated starting from an M0 (i.e., all non-input signal nodes set to zero) cellular environment and switching on all input nodes, as reported in literature. Following the typical cellular response to inflammation, starting from an M1-like configuration, and M2-related external stimuli (i.e., IL-4 for M2a, IL-10 for M2c and IC in combination with LPS or IL-1β for M2b macrophages), the dynamics of transcription factors and secreted molecules (i.e., IL-12 and IL-10) show the macrophage moves from pro- to anti-inflammatory states, as reported in literature. The M2-related polarizations from an M0 initial state have been also performed to check the ability of the system to simulate the situation in which new monocyte-derived macrophage populations are recruited to the inflammation site during the resolution of inflammation, in addition to M2 macrophages polarized from the pro-inflammatory M1 state (see Supplementary File S2).

We also tested *in silico* the “plasticity” of the polarized phenotypes, i.e., the capability to revert the state from inflammatory to anti-inflammatory and *vice versa*. In order to proceed, we run a set of numerical experiments in which macrophages, starting from the four polarized states M1, M2a, M2b, and M2c, were challenged with the four characteristic stimuli (i.e., pro-M1, -M2a, -M2b, and -M2c) resulting in 16 possible couples “initial condition/stimuli.” Each of those simulation settings was repeated 10^4^ times using the asynchronous updating scheme and averages were computed. After that, we used the steady states obtained as initial states for other simulations, giving each input from the input set (see Figure 5).

We focused on M1-related initial states, since a normal immune response begins with an inflammation state, followed by anti-inflammatory environment settings.

With an M0 steady state as initial condition, several stimuli were applied for each simulation. To represent the M1 polarization we gave a combination of random M1-related stimuli (LPS, GM-CSF, IFN-γ, and IL-1β). The initial state for each node of the network are those related to the M0 steady state (no active nodes at all).

We then performed M2a, M2b, and M2c polarizations with IL-4, a combination of IC and IL-1β or LPS, and IL-10 as inputs, respectively. In other words, we started with M1 macrophages, changed their environment and stimulated them with different types of stimuli. Thus, we performed all the combinations for the simulations and analyzed the dynamics and the differences (see Figure 5 for details). We also investigated the possibility of transforming an M2-like phenotype to an M1 macrophage by changing the environment using a variety of external stimuli. However, all considered combinations resulted in states that do not characterize the macrophage M1 canonical phenotype.

### Robustness Evaluation of the Macrophage Network

Biological networks are considered to be robust when compared to random networks, if a single perturbation does not influence the behavior of the entire system. We analyzed the robustness of macrophage polarization network as follows. First, we evaluated the *transition robustness* by perturbing states of the network with random bit flips (Müssel et al., 2010). When the successor states of the original and the perturbed states are computed, the distance between then is calculated as the Hamming distance (HD, that is, the difference between strings of equal length is the number of positions at which the corresponding symbols are different). The HD, normalized by the number of genes in the network, shows how robust the network is to small mutations: the lower the normalized HD, the more robust is the network.

**FIGURE 6:**
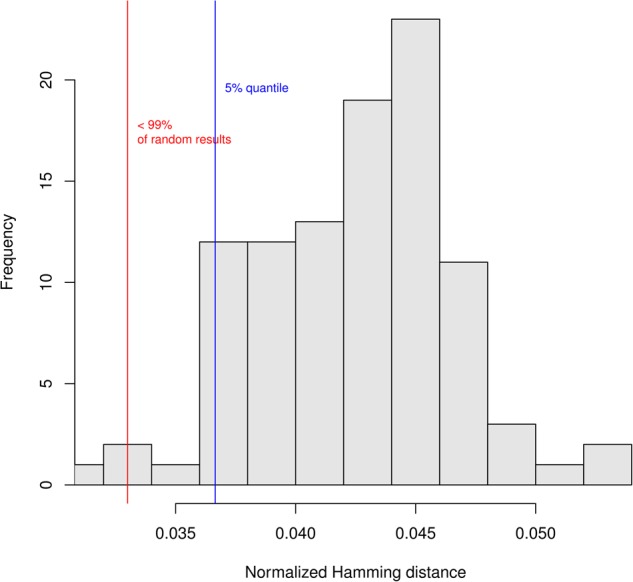
Test of the robustness of the macrophage network. Histogram of the normalized Hamming distance (HD) of randomly generated networks (RGN) in comparison to the HD of the perturbed macrophage network (PMN). The red line shows the mean of the PMN-HD histogram (not shown) which is smaller than the 5% quantile of the RGN-HD distribution (blue line). The test shows that the noise influences the randomly generated networks significantly more than the macrophage network (Müssel et al., 2010).

A hundred of these tests were repeated for 100 randomly generated networks and the results plotted in Figure 6. Results show that the macrophage model is statistically more robust (*p* = 0.01) in comparison to the randomly generated networks. The resulting mean normalized HD equal to 0.03 can be interpreted as if, on average in the mutated networks, 3% of the gene states are different.

### Effects of Knockouts in the Simulations

To analyze the dynamics and investigate the role of each component in the polarization process, we performed knockout (components’ value set to “0”) and ectopic expression (components’ value set to “1”) *in silico* experiments. These constraints allowed us to see how perturbations of the system affect the network functionality with respect to the macrophage behavior. At a biological level, this analysis may have potential impact in in-silico pharmaceutical target prioritization.

In our network, gene knockout is interpreted as a deactivation of one or more components, just like the deactivation of a protein that is a target of a drug.

**FIGURE 7:**
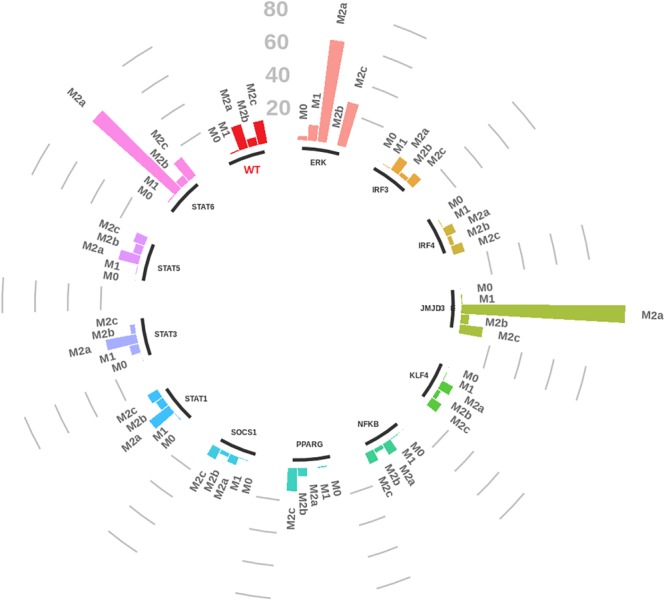
Circular bar plot of macrophage gene knockouts. Each group represents the knockout of a specific transcription factor of the network. Bar heights represent the number of steady states for each macrophage canonical phenotype with respect to the wild type (WT in red).

We performed systematic knockouts on every internal node of the network (internal transducers/transcription factors), to see how they affect the dynamics of the network by calculating the fold change of the number of steady states reached by the system (see Figure 7 and Supplementary Files S3, S4 for details). The idea is that a knockout modifies the network characteristics so that also its dynamics is modified and the number of steady states, for example, a higher number of pro-inflammatory steady states is interpreted as a greater probability to induce, *via* that specific knockout, a pro-inflammatory polarization of the macrophages.

## Discussion

**FIGURE 8:**
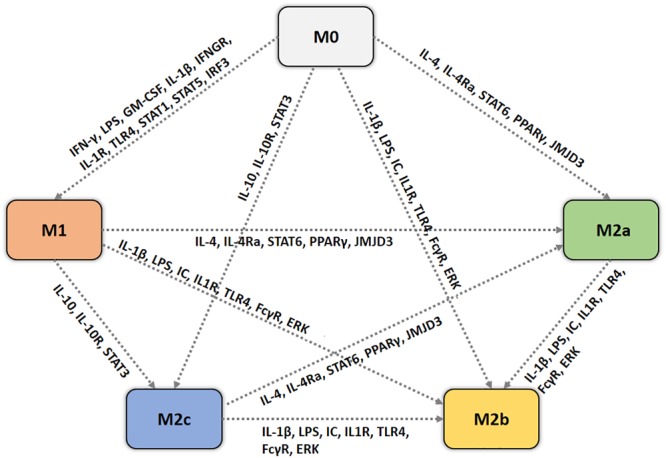
Cell fate map for macrophages. Each dotted arrow represents the switch of macrophage from a phenotype to another, annotated with the gene expression patterns, based on simulation dynamics and results.

Pro-inflammatory macrophages are those polarized by cytokines like IFN-γ or LPS (among other molecules). They are produced during cell-mediated immune responses, interacting with chemical mediators produced by other cells, such as the IFN-γ secreted by natural killer (NK) cells (Mosser and Edwards, 2008). Resting macrophages are primed by IFN-γ to produce pro-inflammatory cytokines, according to our simulations of an unstimulated macrophage which undergoes an M1 polarization when stimulated by IFN-γ (see Figure 5). TLR ligands, such as the well-known LPS can also polarize macrophage into an M1 form, *via* NF-κB signaling, producing pro-inflammatory mediators, other stimuli such as GM-CSF and IL-1β gave similar results (Mosser and Edwards, 2008; Lawrence and Natoli, 2011; Sica and Mantovani, 2012). Macrophages respond to micro-environmental cues, showing a distinct transcriptional profile depending on the stimulus. Starting from M0, that is assumed to be a cell with no typical constitutive gene expression profile, an M1 stimulus (i.e., IFN-γ, LPS, IL-1β, and GM-CSF) leads to a M1 phenotype, IL4 to a M2a phenotype, IC together with LPS and/or IL-1β to an M2b phenotype, and IL-10 to a M2c phenotype, the network can represent the polarization process (see Figure 8 for a visual representation of macrophage switch pathways).

Transcription factor NF-κB is among the most important regulators of M1 polarization of macrophages (Wang et al., 2014). Its expression is stable and maintained during macrophage polarization after stimulation with M1-related inputs. If no inputs are given to an M1-polarized system, NF-κB seems to maintain the M1 polarization (see Figure 5), while STAT1 and STAT5 decrease their expression (if not stimulated by IFN-γ and GM-CSF), until an M2-related stimulus (IL-4, IL-10 or IC) is present, which result in the resolution of the inflammation phase, and in the increase of the expression of M2 master regulators.

In the presence of IL-4 (i.e., activation of input node IL4), we noticed rapid expression of M2a master regulators (i.e., STAT6, PPARγ, and JMJD3) and the production of IL-10, with a slow decrease in the production of IL-12, indicating that M2a-related stimuli can immediately suppress the pro-inflammatory function of macrophage, as already evidenced in literature (Sica and Mantovani, 2012). In M2b polarization, despite the slow decrease of the expression of pro-inflammatory transcription factors and secreted molecules, IL-10 is finally produced by this type of macrophage, and its master regulator, ERK. M2c polarization is reached when IL-10 is given as input, with IL-10 production and STAT3 expression.

In the absence of external stimuli, a polarized M2 macrophage maintained its state with no alteration on the molecules expression, highlighting the stability of this phenotype.

M1 stimuli do not affect M2-like macrophage, apart from M2b in which we can assist to a slower decrease of IL12, reaching its stable state at the seventh time step, at variance with M2a and M2c simulations in which the anti-inflammatory stimuli lead to the absence of IL12 at the fourth time step. For any input given to an M2b-polarized macrophage, a phenotype change related to the given stimulus seems to be a common feature, except for M1 stimuli, which appear to polarize macrophage to a form corresponding to the production of both output cytokines (IL12 and IL10) and the repression of ERK. This behavior has not been reported in literature, but could explain the existence of this not-well characterized type of macrophage that share common features between pro- and anti-inflammatory macrophages (Sica and Mantovani, 2012).

A similar behavior can be observed when M2c macrophage are polarized with M1-related cytokines, even though M2a and M2b stimulations can subvert M2c polarization, indicating that M2c macrophages are more likely to be polarized from an M0 phenotype or switch from an already M1-polarized macrophage. Indeed, in some physiological and pathological conditions, such as muscle regeneration, the co-existence of different populations of M2 macrophages can be found at later stages, comprising M2a and M2c macrophage (Novak et al., 2014; Rigamonti et al., 2014). Hence, they can be thought of as distinct populations of macrophage polarized independently, since this regulatory network is characterized by well-known interactions between molecules involved in the polarization pathway (Novak et al., 2014; Rigamonti et al., 2014).

## Conclusion

Transforming acute diseases into chronic ones is a realistic strategy for those pathologies for which no definitive cure is known, such as in the case of HIV (Scandlyn, 2000). A better understanding of the pathways involved in the transition from acute to chronic states and a more comprehensive knowledge of the cellular and molecular mechanisms are in need. Understanding how the immune response is regulated, and how immune cells integrate information from the multitude of molecular signals could certainly lead to improvements of existing therapies and make suggestions on the way forward.

In this work, we presented a dynamic logical model of the GRN of macrophage polarization, which is coherent to the expected behavior, under different experimental conditions. The model identified mechanisms driving a pro- into an anti-inflammatory setting, and hence maybe useful in transforming, fully or in part, an acute inflammation into a chronic one.

One example of network dynamics that could be affected by providing different types of stimuli is reported in Figure 8. We examined the different dynamics of this process to study how macrophages switch their phenotype during ineffective and sterile immune responses, focusing on M2-like polarization from a pro-inflammatory micro-environment.

A first result regards the importance of two inhibitions, namely, of TLR4 and NF-κB signaling by FcγR, that turned out *essential* to obtain the M2b phenotype. In fact, a preliminary version of the network, not accounting for these two inhibitions, was not able to reach the M2b polarized state.

The repolarization from M2 to M1 has been experimentally observed, yet occasionally in specific environments (Davis et al., 2013; Zheng et al., 2013; Zhang et al., 2017; Gao et al., 2018). Simulation results suggest that such polarization reversion seems to show a higher inertia. In fact, as shown in Figure 5 panels a, b, and c, the average values of pro-inflammatory genes starting from an anti-inflammatory phenotype only reach the value of 30% of the activation level. Furthermore, our *in silico* knockout experiments evidenced how some regulator plays a role by downregulating genes that are known for their inhibition activity. For instance, in M2-related knockouts *in silico* experiments, such regulators, as for example PPARG, are responsible for the resolution of inflammation and the maintenance of an anti-inflammatory environment by enabling the production of IL-10 and other important anti-inflammatory mediators. Similar studies could focus on networks that are specific to some pathogen or some physiological mechanism, to get a better comprehension in terms of the logic of the regulatory machinery.

This modeling study yielded another important observation, which is related to the environmental-dependent expression of mixed markers identifying one of the four canonical macrophage polarizations. Indeed, recent studies support the view that fully polarized macrophages (M1 and M2) as being the extremes of a continuum of macrophages polarization (Mantovani, 2008). This could for example be obtained by mixing various stimuli, such as IC together with LPS or IL-1β and IL-10, which give rise to M2-like functional phenotypes, yet sharing properties with IL-4-activated macrophages (Mantovani et al., 2004). This continuum of macrophages phenotypes parallels a continuum in CD4+ T cell states, recently observed, as opposed to a limited number of discrete phenotypes (Eizenberg-Magar et al., 2017). Indeed, while T helper cell induction requires the participation of macrophages, several signal feedback mechanisms are implemented for the activation and differentiation of macrophages. Even if this intertwinement may vary in both quantitative and qualitative aspects, the continuum of states detected in T helper and macrophage cells may be more linked than observed up to now.

**FIGURE 9:**
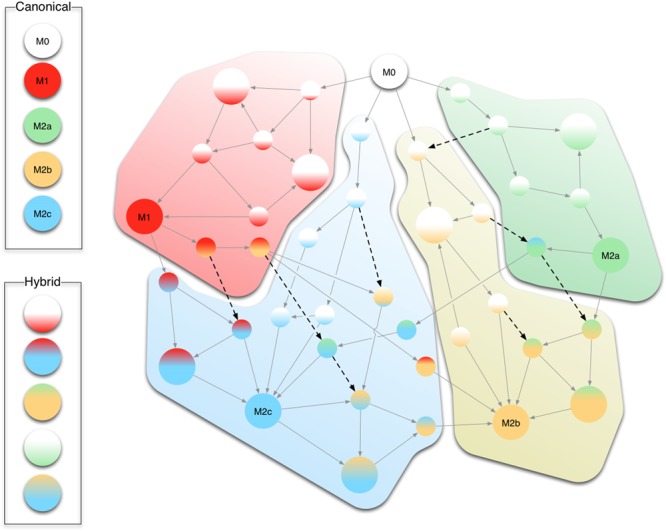
Conceptual representation of the continuum of differentiation states. Circles show intermediate stable states (smaller circles) between the five canonical M0, M1, and M2a/b/c (larger monochromatic circles). Stable states whose correspondent phenotype is not uniquely determined are indicated as larger circles with more than one color. Gray arrows indicate state changes the cell undergoes upon reception of extracellular stimuli. Black dashed arrows show jumps from one differentiation pathway to another. For instance, just by changing the extracellular stimuli (e.g., IL10) a macrophage which started the differentiation from M1 to M2b can divert toward the M2c phenotype.

We surmise that shifts among different phenotypes in our model mimic the hypothetical continuum of macrophage polarization, being M1 and the three subtypes of M2 the extremes of such uninterrupted sequences of states. Figure 9 conceptualizes this continuum in the progression of gene activations leading from one form of polarization to another driven by various stimuli. For instance, an M1+M2 successive stimuli can lead to an M2a stable configuration while passing through an M1 state (see Figure 9).

The presented approach, although promising and general, is not free of pitfalls. Even if little mathematical knowledge is needed to build a Boolean network, the information gained from its analysis is strongly affected by the accuracy of the relationships among genes encoded in the Boolean rules characterizing the overall dynamics. Manually curated networks optimally convey the biological information but cannot ensure completeness. The usefulness of Boolean networks therefore is found while dealing with poorly characterized systems, especially when quantitative experimental data is missing. In some cases, alternative approaches should be considered such as introducing uncertainty with probabilistic networks or using continuous models that describe the kinetic with greater accuracy than Boolean networks.

To conclude, although there is a wealth of information about the different macrophage subsets *in vitro*, features such as plasticity, heterogeneity, and adaptability make them very difficult to study using conventional experimental tools. In this paper, we have shown that relatively simple logical description of the gene regulation machinery can support the analysis of the emerging complexity of the phenomena of mammalian cell differentiation and can be used to provide testable predictions as, for instance, which combination of stimuli leads to hybrid phenotypes.

The network provided here is manually curated and has been built based on the available information derived from literature to date. This should be considered as-is, that is, limited to the current knowledge which, regarding the less characterized pathways and molecular interactions leading to M2b macrophages, is admittedly lacking.

## Author Contributions

All authors conceived the study. AP and FC performed the experiments. All authors carried out the analysis and contributed to writing the paper.

## Conflict of Interest Statement

The authors declare that the research was conducted in the absence of any commercial or financial relationships that could be construed as a potential conflict of interest.

## Footnotes

1 www.ginsim.org
