# Supplementary figures and images for: Gene Regulatory Network Modeling of Macrophage Differentiation Corroborates the Continuum Hypothesis of Polarization States
Source: Front Physiol. 2018 Nov 27;9:1659. doi: 10.3389/fphys.2018.01659 (PMC6278720; doi:10.3389/fphys.2018.01659)

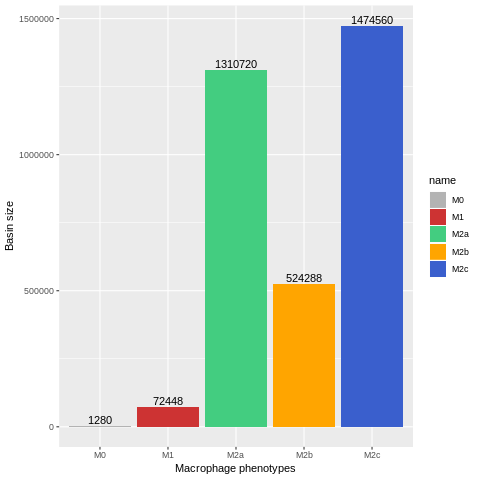

Supplement: Supplementary file 4 [file Data_Sheet_4.ZIP › S4_barplots_of_basin_sizes/IRF4_KO_basin.png]

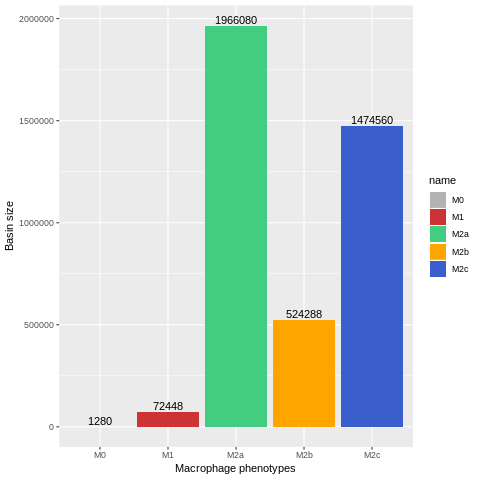

Supplement: Supplementary file 4 [file Data_Sheet_4.ZIP › S4_barplots_of_basin_sizes/IRF3_KO_basin.png]

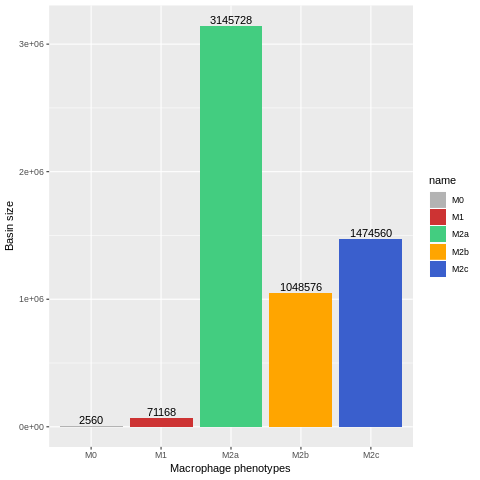

Supplement: Supplementary file 4 [file Data_Sheet_4.ZIP › S4_barplots_of_basin_sizes/STAT1_KO_basin.png]

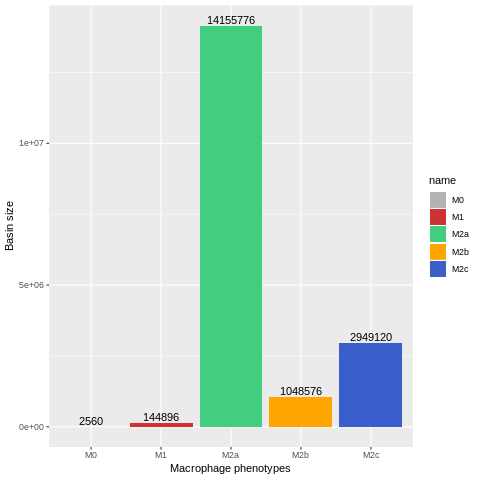

Supplement: Supplementary file 4 [file Data_Sheet_4.ZIP › S4_barplots_of_basin_sizes/STAT6_KO_basin.png]

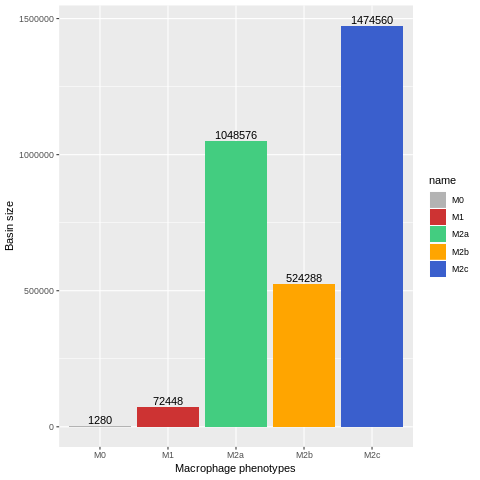

Supplement: Supplementary file 4 [file Data_Sheet_4.ZIP › S4_barplots_of_basin_sizes/SOCS1_KO_basin.png]

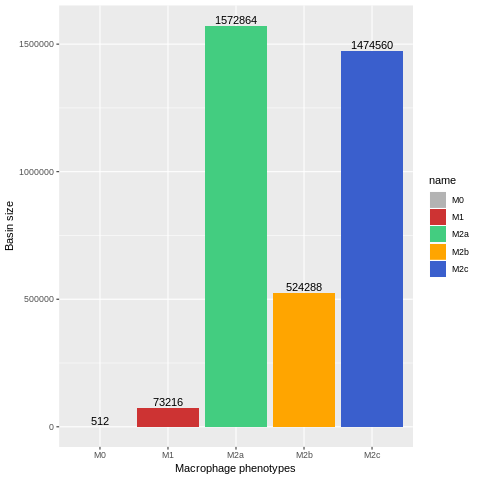

Supplement: Supplementary file 4 [file Data_Sheet_4.ZIP › S4_barplots_of_basin_sizes/KLF4_KO_basin.png]

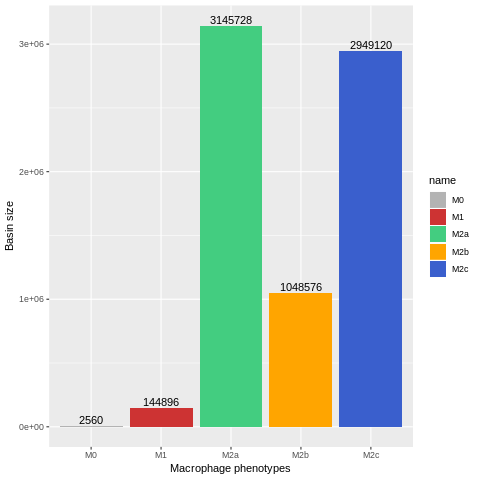

Supplement: Supplementary file 4 [file Data_Sheet_4.ZIP › S4_barplots_of_basin_sizes/WT_basin.png]

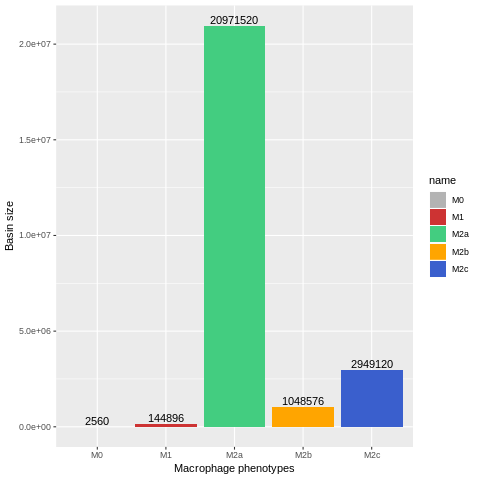

Supplement: Supplementary file 4 [file Data_Sheet_4.ZIP › S4_barplots_of_basin_sizes/JMJD3_KO_basin.png]

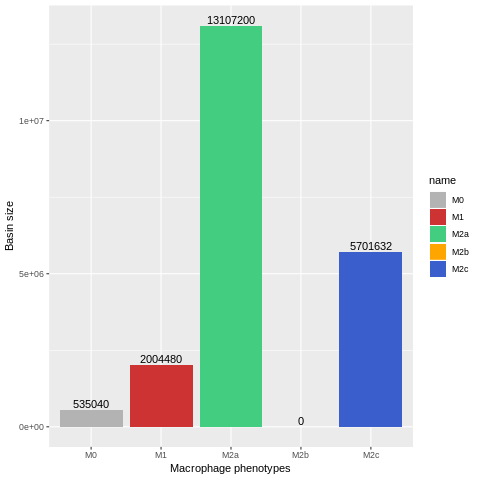

Supplement: Supplementary file 4 [file Data_Sheet_4.ZIP › S4_barplots_of_basin_sizes/ERK_KO_basin.png]

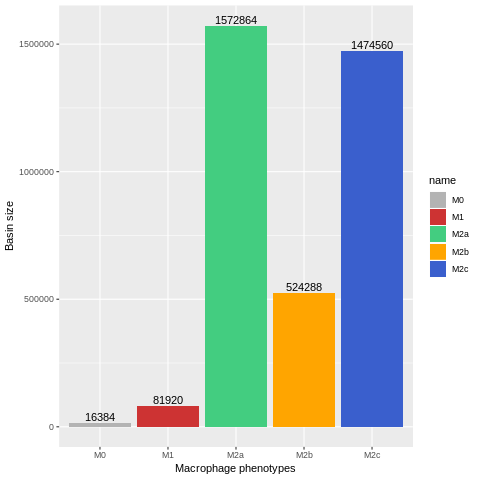

Supplement: Supplementary file 4 [file Data_Sheet_4.ZIP › S4_barplots_of_basin_sizes/NF_kB_KO_basin.png]

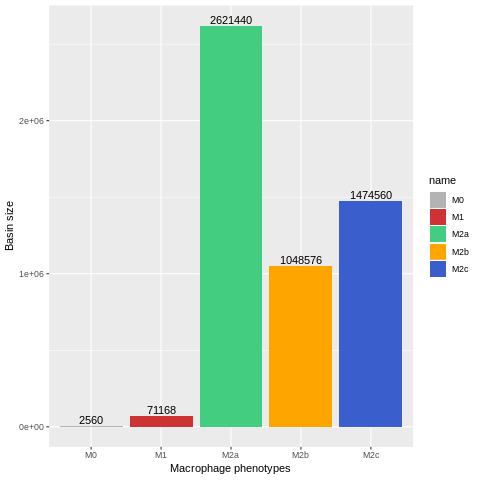

Supplement: Supplementary file 4 [file Data_Sheet_4.ZIP › S4_barplots_of_basin_sizes/STAT5_KO_basin.png]

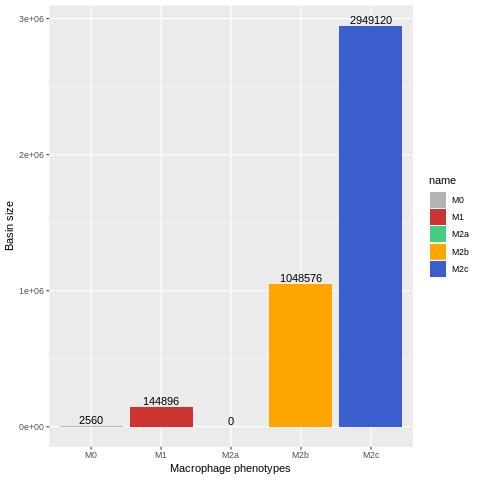

Supplement: Supplementary file 4 [file Data_Sheet_4.ZIP › S4_barplots_of_basin_sizes/PPARg_KO_basin.png]

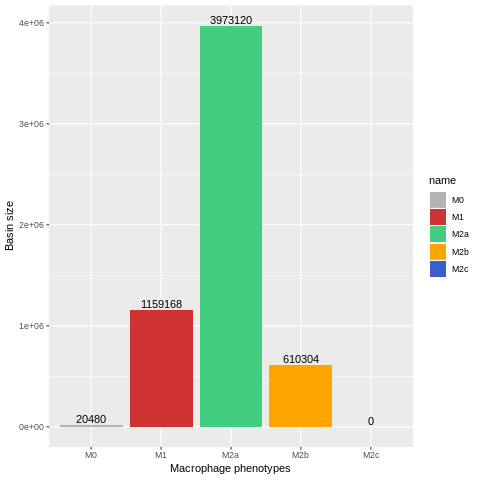

Supplement: Supplementary file 4 [file Data_Sheet_4.ZIP › S4_barplots_of_basin_sizes/STAT3_KO_basin.png]
